# A Field Evaluation of the LuciTrap and the Western Australian Trap with Three Different Baits Types for Monitoring *Lucilia cuprina* and *Lucilia sericata* in New Zealand

**DOI:** 10.3390/insects12090829

**Published:** 2021-09-15

**Authors:** Paul Brett, Kevin Lawrence, Paul Kenyon, Kristene Gedye, William Pomroy

**Affiliations:** 1 School of Veterinary Science, Massey University, Private Bag 11-222, Palmerston North 4442, New Zealand; K.Lawrence@massey.ac.nz (K.L.); K.Gedye@massey.ac.nz (K.G.); w.pomroy@massey.ac.nz (W.P.); 2 School of Agriculture and Environment, Massey University, Private Bag 11-222, Palmerston North 4442, New Zealand; p.r.kenyon@massey.ac.nz

**Keywords:** *Lucilia cuprina*, *Lucilia sericata*, flytraps

## Abstract

**Simple Summary:**

In New Zealand, flystrike is caused by two Dipteran species, *Lucilia cuprina* and *Lucilia sericata*. This study contrasts four flytrap treatments, the LuciTrap with its combination of three chemical lures (Lucilures) and the Western Australian Trap with three different bait types (LuciLure, Sheep liver with 30% sodium sulphide and squid) during mid-summer. The aim of the study is to assess the most effective trap and bait combination that New Zealand farmers may use on their farms to monitor *L. cuprina* and *L. sericata*. This study found that either the LuciTrap or the Western Australian Trap with sheep liver and 30% sodium sulphide were the most effective traps to catch *L. cuprina* and *L. sericata*.

**Abstract:**

Flytraps can be used on farms to monitor the populations of primary strike flies (*Lucilia cuprina* and *Lucilia sericata*) and, hence, offer a view regarding the incidence of flystrike on sheep. This study aimed to contrast the specificity and effectiveness of the LuciTrap with its combination of three chemical lures (Lucilures) and the Western Australian Trap with three bait types (LuciLure, Sheep liver with 30% sodium sulphide and squid). A mean model and rate model were fitted to the data. The mean model showed no difference (*p* > 0.05) in the mean weekly catch for *L. cuprina* between the Western Australian Trap with LuciLures and the Western Australian Trap baited with sheep liver with 30% sodium sulphide (*p* < 0.05). Whereas, for *L. sericata*, no difference (*p* > 0.05) was found between the Western Australian Trap with LuciLures, the Western Australian Trap baited with sheep liver with 30% sodium sulphide and the LuciTrap. The rate model illustrated that the Western Australian Trap with sheep liver with 30% sodium sulphide and LuciTrap did not differ (*p* > 0.05) for *L. cuprina* and *L. sericata*. Combined, these results indicate that New Zealand farmers can use either the LuciTrap or the Western Australian Trap with sheep liver with 30% sodium sulphide to monitor these target species.

## 1. Introduction

Flystrike or cutaneous myiasis is a disease caused by certain species of blowflies that lay eggs on the skin surface of sheep with larvae that subsequently parasitise the skin surface. If left untreated, a sheep can be subject to repeated oviposition; this can result in distress to the animal and can lead to the death of the animal. In New Zealand, three species of Diptera can cause flystrike in the absence of open wounds: *Lucilia cuprina* and *Lucilia sericata* are regarded as the main species, while *Calliphora stygia* is associated with only a few cases [1,2].

Flytraps can be utilised on sheep farms for two main purposes. The first use is to reduce the populations of *Lucilia* and, thus, the incidence of flystrike on sheep [3,4]. The second use is as a surveillance tool, enabling farmers to recognise when myiasis-inducing flies are present on their farms, i.e., to signal the start of a new flystrike season and make informed strategic management decisions to control flystrike [4]. It was shown that, in the United Kingdom, the deployment of flytraps in early spring reduced the emerging population of primary strike flies and reduced the annual flystrike levels [3]. However, it is shown that the use of flytraps did not reduce the number of cases of flystrike on a farm in New Zealand during the course of a season [4].

Numerous types of flytraps have been trialled and used on farms, including bait bins [5], the Western Australian Trap [6,7,8], a wind-orientated Western Australian Trap [9,10,11], water-based traps, such as the Rescue Trap^®^, Red Top Flycatcher^®^, the Buzz Disposable Flycatch^®^ and Easy trap^®^ [12,13] and the LuciTrap^®^ [14,15,16,17]. The LuciTrap includes three chemical lures (LuciLures), which are held in plastic containers within the body of the LuciTrap. The LuciLure containers have open cotton wicks to dispense chemicals and last for up to six months.

Although there have been some contrasting results, overall, the LuciTrap has been shown to be the most effective trap for *L. cuprina* in Australia [17] and South Africa [18]. Although the effectiveness of the LuciTrap for *L. cuprina* in New Zealand is currently unknown, it appears to be the most appropriate method for trapping *L. cuprina* due to the specificity of its trapping design. Two important features of the trap design are the attractiveness and persistence of the chemical lures and the size of the holes in the lid, which restrict the entry of non-target flies [19].

In contrast, there have been differing reports of the success of the LuciTrap for catching *L. sericata*, performing poorly in Hungary [20] compared to South Africa [18] and Australia [17,21]. In the Hungarian study, it was theorised that the holes in the lid of the LuciTrap were too small for *L. sericata* to enter the trap itself, as flies were observed around the periphery of the LuciTrap but not inside the trap itself [20]. That being said, alternative lures and not the three LuciLures from the LuciTrap were used in the Hungarian study, which may account for the differences found in that study compared to those in South Africa and Australia.

The Western Australian Trap design has been widely used in several studies in Australia to trap *L. cuprina* and *L. sericata* using sheep liver and sodium sulphide as bait [7,22]. More recently a modified version of the Western Australian Trap constructed from easily obtained plastic containers has been used extensively in studies across New Zealand [8]. The overall efficiency of the Western Australian Trap is unknown, although previous studies using this trap have reported that it is effective in trapping all target species [8].

Sheep liver in 30% sodium sulphide has historically been used as bait to successfully attract necrophagous flies. The addition of sodium sulphide prevents desiccation of the liver itself, as well as acting as a chemical attractant in its own right. However, one of the major drawbacks of this bait is the need to replace it on a fortnightly basis [23]. Furthermore, there are health and safety concerns about the use of sodium sulphide in New Zealand at present and access to the chemical for farmers to self-dispense is restricted, owing to the dangers it poses to people and the wider environment [24].

It is unknown if substituting the liver in sodium sulphide with an alternative offal bait or with the LuciLures in the Western Australian Trap could be as effective in attracting and monitoring a variety of necrophagous flies, although the use of LuciLures themselves have some restrictions. One alternative bait is squid, which was found to be effective in attracting *L. sericata* and other blowflies [25]. Further potential benefits of squid are that it does not require the addition of chemicals and is readily available through fishing tackle outlets.

However, the effectiveness of squid for monitoring *L. sericata* and *L. cuprina* when placed in the Western Australian Trap is unknown under New Zealand conditions. Therefore, this study aimed to contrast the effectiveness and specificity of the catch of LuciTrap with its lures and the Western Australian Trap using three types of lures (LuciLures, squid and sheep liver with 30% sodium sulphide). The overall aim of this study is to provide farmers with a trap and bait combination that may be used to monitor the populations of the main species and, therefore, be used as a means to actively manage flystrike on farms.

## 2. Materials and Methods

This was an incomplete factorial experimental design in which two types of traps: the LuciTrap (Bugs for Bugs, Toowoomba, Queensland, Australia) and the Western Australia Trap as modified by Cole [8] and three types of baits (LuciLure, Bugs for Bugs, Toowoomba, Queensland, Australia) comprising three separate chemical lures (LuciLure A—120 g/L sodium sulphide—80 mL; LuciLure B—1055 g/L 2-mercaptoethanol at 95%, 47 g/L indole—60 mL at 5%; and LuciLure C—960 g/L butanoic acid, 60 mL held in separate plastic containers with cotton wicks), sheep liver (50 g in 100 mL of 30% sodium sulphide) and squid (50 g) were used.

Therefore, the four treatments compared in this study were: (i) LuciTrap with its three LuciLure baits (Luci), (ii) Western Australian Trap with squid (WAT (Sq)), (iii) Western Australian Trap with the three LuciLure baits (WAT (Luci)) and (iv) the Western Australian Trap with sheep liver and 30% sodium sulphide (WAT (LivSS)) as presented in Table 1.

**Figure 1:**
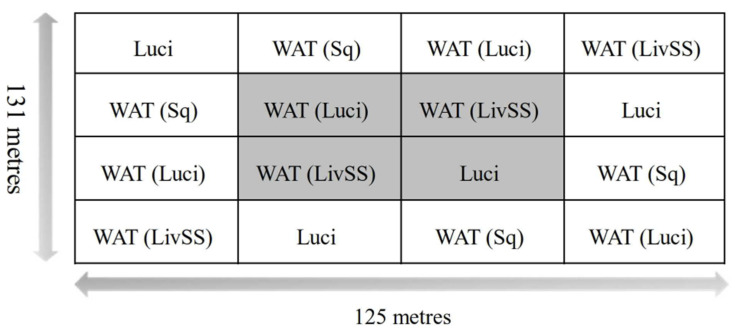
Outline of the four-by-four orthogonal Latin square array study design. LuciTrap with LuciLure A, B and C (Luci); Western Australian Trap with squid (WAT (Sq)), Western Australian Trap with LuciLure A, B and C (WAT (Luci)); and Western Australian Trap with sheep liver and sodium sulphide (WAT (LivSS)). Grey shading designates the inner portion, while the white background designates the outer portion of the trapping study design.

The study was conducted on Massey University’s Keebles Sheep Farm approximately 5 km from Palmerston North at Massey University in New Zealand (40°23′30.1″ S latitude, 175°36′18.3″ E longitude). Throughout the study, sheep were grazing in the paddocks adjacent to where the traps were located. A total of sixteen traps and bait combinations, with four replications of each treatment. The traps were placed 1.5 m above ground and arranged in a four-by-four orthogonal Latin square array with each equally spaced (Figure 1). The study was conducted over six weeks in mid to late summer (42 days from the 18 January until the 8 March 2019), and each trap remained in the same position throughout the study.

Weather data was sourced from Palmerston North Airport (40°19′15.6″ S latitude, 175°37′05.7″ E longitude) managed by the National Meteorological Service (https://cliflo.niwa.co.nz/, accessed on 1 October 2019) approximately 8 km from the study site.

A modified version of the Western Australian Trap as previously described [8] was utilised in this study, with the following alterations. The trap was constructed from two 3.2 L clear polystyrene plastic domestic storage containers (Click Clack, Innova Products Limited, Palmerston North, New Zealand) sitting upside down on top of each other. Eight 10 mm diameter holes were made around the lower chamber of the trap.

**Figure 2:**
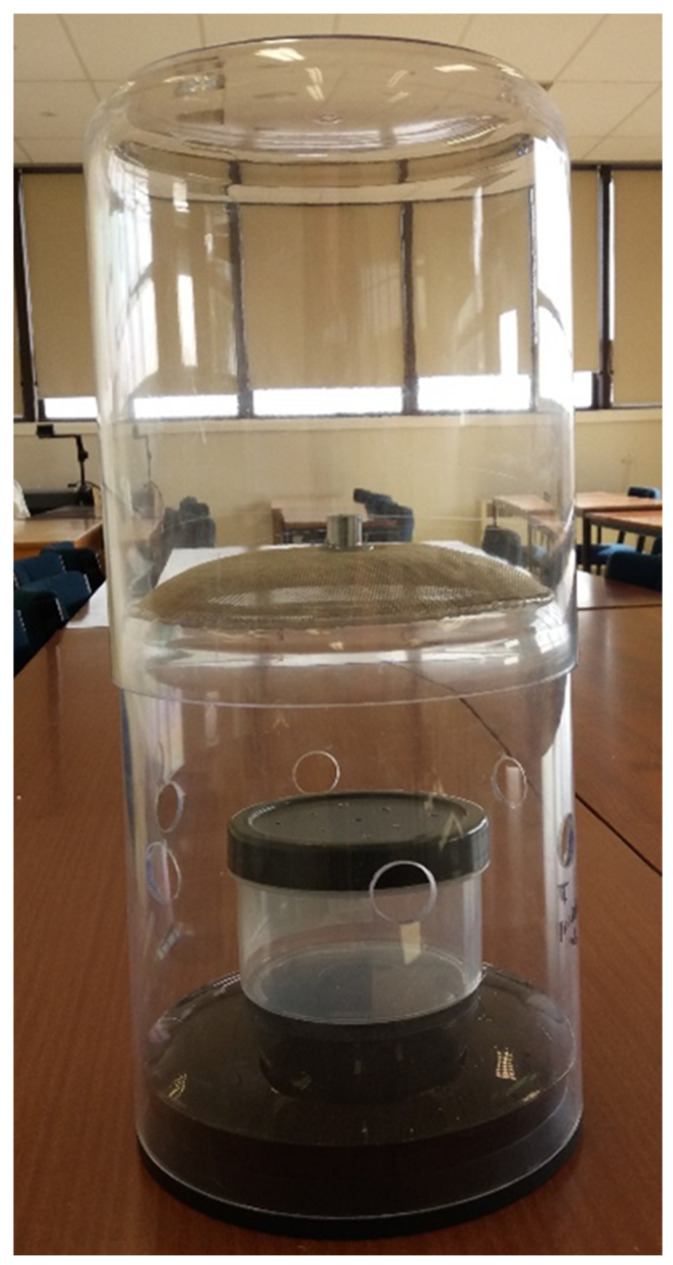
The unpainted version of the Western Australian Trap, showing the internal design.

**Figure 3:**
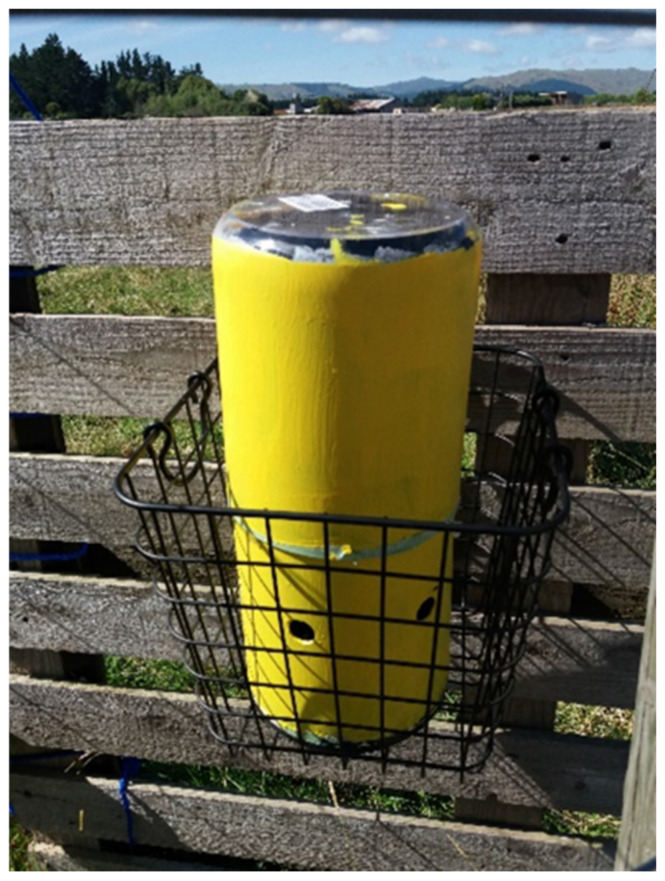
Painted Western Australian Trap in a wire basket at the study site.

A metal gauze was fashioned into a bowl shape with its convex side facing upwards and then glued to the inside of the upper chamber about 200 mm from its lower margin to facilitate the upper sections sitting on the lower section. A 10 mm diameter metal tube was placed in the centre of the gauze for flies to pass through (Figure 2). The inner and outer layers of both chambers were first painted with a waterborne surface sealer; then, the outer surface was painted bright yellow and the inside painted black (Figure 3). The top of the upper chamber was left unpainted, as flies would enter the trap and naturally move towards a light source, thus, remaining in the trap.

For the treatments, WAT (Sq) and WAT (LivSS), the squid and the 50 g of sheep liver in 100 mL of 30% sodium sulphide, respectively, were placed in a 250 mL plastic container with a plastic lid punctured with 1–2 mm holes in the lower chamber (Figure 3). The LuciTrap lures in the WAT (Luci) were taped together in an upright position in the lower chamber. Each Western Australian trap was placed in a metal wire basket to keep it upright throughout the study and to prevent any obstruction of the entry holes (Figure 3). The LuciTraps were bolted to a secure post.

All traps were sampled on a weekly basis, and the flies collected were preserved in 70% alcohol. The day of collection was the first day of the collection week. The 50 g of sheep liver in 100 mL of 30% sodium sulphide and squid were individually replaced on a weekly basis. The LuciLure baits were not changed in the LuciTrap nor the Western Australian Trap for the six-week duration of the study.

### 2.1. Identification 

Collected flies were identified using a stereomicroscope. Muscidae and Sarcophagidae were identified to the family level, whilst all Calliphoridae were identified to species level using published keys [25,26,27,28,29,30,31].

### 2.2. Statistical Analysis

The distribution of trapped flies was presumed to be from a Poisson process. However, exploratory plots also showed that the data for both *L. cuprina* and *L. sericata* were zero-inflated. Consequently, the following series of nested models were tested to identify the best fit for the data: Poisson, Quasi-Poisson, negative binomial, zero-inflated Poisson, zero-inflated Quasi-Poisson and zero-inflated negative binomial, using the *glmmTMB* package [32] in R [33].

Two count models were fitted to the data for each of our target species, the first was the mean model of the mean catch per week for each trap type:

(1) $$\ln\left(\mathrm{target}\;\mathrm{species}\;\mathrm{catch}\right)={\;b}_{0}+b_{1}x_{1}+\ldots +{\;b}_{k}x_{k},$$

And the second was a rate model, which was the mean model adjusted for the total catch per week for each target species and trap type:

(2) $$\ln\left(\mathrm{target}\;\mathrm{species}\;\mathrm{catch}\right)={\;b}_{0}+b_{1}x_{1}+\ldots +{\;b}_{k}x_{k}+\ln\left(\mathrm{total}\;\mathrm{catch}\right)$$

where $\ln\left(\mathrm{total}\;\mathrm{catch}\right)$ in Equation (2) is referred to as the offset and the beta coefficient is constrained to equal 1, so that the estimated rate is the true rate. The rate estimated using this model is the number of target species caught per 100 total flies caught.

These two models provide different information, the first model identifies which trap and bait combination caught the most target species flies per se, whilst the second indicates which trap and bait combination was more discriminating towards each target species. A more discriminating trap could potentially make it far easier for the farmer to observe the target species when they first appeared in the season. In the models, each of the traps was compared to the WAT (LivSS).

The week of the collection was entered as a continuous variable a priori into all of the models. In addition, individual weather variables per week (the mean temperature, minimum temperature, maximum temperature, total rainfall and mean wind speed) were tested and retained if at *p* < 0.05. However, due to the potential of temperature variables being co-linear to each other, only one temperature variable was tested in the model at a time. Variables were removed using a backward selection method, and interactions between weather variables and trap type were also tested in the model.

Each model was compared using Akaike’s information criterion (AIC) and Bayesian information criterion (BIC) [34]. The model with the lowest AIC and/or BIC was considered to be the best fitting model for the data. The *emmeans* package [35] was used to compare the estimated mean catch and the rate of catch for each trap type for *L. cuprina* and *L. sericata*. A Tukey’s comparison was then made to compare trap types using *p* < 0.05. The simulated residuals of the resulting models were then compared to the observed data using the *DHARMa* package [36] to assess the fit of the models.

The mean daily temperature/week was calculated over seven days, and the rainfall was calculated as the total rainfall per week. The maximum and minimum temperatures were taken as the maximum and minimum temperatures recorded at any time over the course of the entire week. The mean wind speed was calculated as the mean speed over seven days.

To assess the potential influence of a location of a trap and bait combination in the Latin square, each trap was designated as being either an inner or an outer trap (Figure 1). The catch from each location, whether inner or outer, was added together for *L. cuprina*, *L. sericata* and by-catch, respectively. The by-catch was designated to be all other non-target species, i.e., not *L. cuprina*, *L. sericata* or *C. stygia*. A one-way ANOVA was then performed to assess whether there was a significant difference between fly catches in the inner and outer traps of the design.

## 3. Results

A total of 22,616 flies were collected over the six weeks (Table 2). This included the following species of Calliphoridae: *L. cuprina*, *L. sericata*, *C. stygia*, *Calliphora quadrimaculata*, *Chrysomya rufificies* and *Chrysomya megacephala*. The two most common calliphorid species caught were *L. cuprina* and *L. sericata* comprising 1.6% and 0.8%, respectively, of the total number of flies caught as shown in Table 2. The most numerous Dipteran families in the bycatch were Sarcophagidae with 80.7% and Muscidae with 16.6% of the total as shown in Table 2.

As indicated earlier, *L. cuprina*, *L. sericata* and *C. stygia* were the three target species. However, a large disparity in the results for each of these species was found. For *L. cuprina* and *L. sericata,* there were many individual traps each week with null catches: *L. cuprina* (31/96, 32%); and *L. sericata* (46/96, 48%) and for *C. stygia*, a total of only three specimens were caught over the entire six-week study, across all trap types. As a result, no further inferences could be made regarding *C. stygia* due to the low sample size.

An overall comparison of the unadjusted catch, based on the confidence intervals, indicates the WAT (Luci) caught more bycatch than Luci (Table 3). All other treatments did not differ for by-catch. WAT (Sq) caught less *L. sericata* than all other treatments, which did not differ from each other. WAT (Sq) caught less *L. cuprina* than WAT Luci and WAT (LivSS), but it did not differ from Luci. WAT (LivSS), WAT (Luci) and Luci did not differ in their weekly catch of *L. cuprina*. There was no effect (*p* > 0.05) on the location of a trap on the inner and outer realms of the Latin square array for *L. cuprina*, *L. sericata* and by-catch.

**Figure 4:**
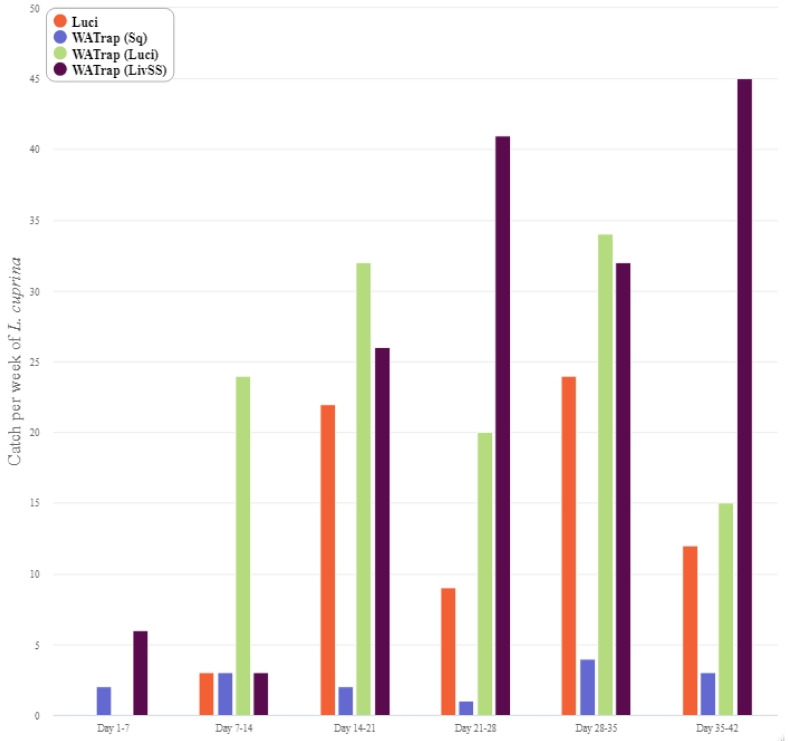
Weekly catch of *Lucilia cuprina* using the four different trap types: LuciTrap with LuciLures A, B and C, (Luci); Western Australian Trap with squid, (WAT (Sq)); Western Australian Trap with LuciLures A, B and C, (WAT (Luci)); and Western Australian Trap with Sodium Sulphide and sheep liver, (WAT (LivSS)).

**Figure 5:**
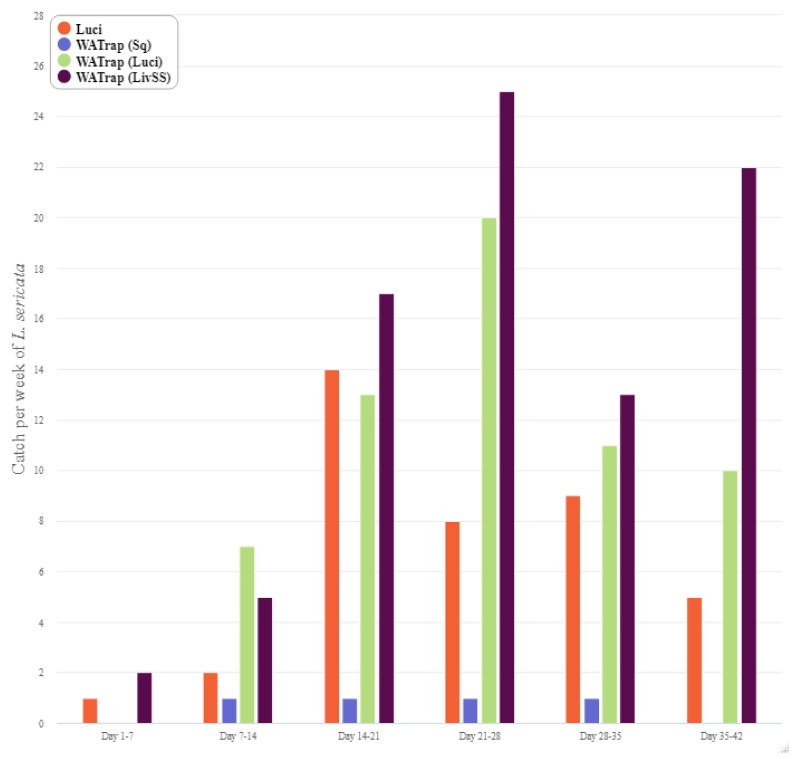
Weekly catch of *Lucilia sericata* using the four different trap types: LuciTrap with LuciLures A, B and C, (Luci); Western Australian Trap with squid, (WAT (Sq)); Western Australian Trap with LuciLures A, B and C, (WAT (Luci)); and Western Australian Trap with Sodium Sulphide and sheep liver, (WAT (LivSS)).

**Figure 6:**
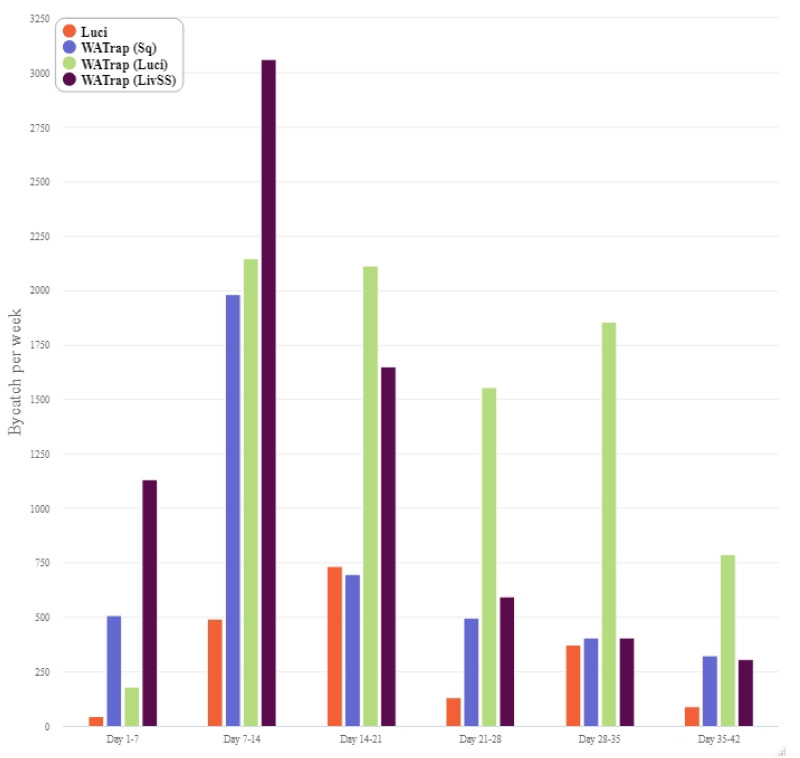
Weekly bycatch with each of the four different trap types: LuciTrap with LuciLures A, B and C, (Luci); Western Australian Trap with squid, (WAT (Sq)); Western Australian Trap with LuciLures A, B and C, (WAT (Luci)); and Western Australian Trap with Sodium Sulphide and sheep liver, (WAT (LivSS)).

The general trend indicates that the catch of *L. cuprina* steadily increased until the fifth week of the study (Figure 4), whereas the catch of *L. sericata* generally peaked in the fourth week of the study (Figure 5). By the third week of the study, the catch from the Luci, WAT (Luci) and WAT (LivSS) treatments increased for *L. cuprina* and *L. sericata,* whereas there was a consistent low catch rate from the WAT (Sq) for both species over the course of the entire study (Figure 4 and Figure 5). Most of the by-catch was in the second week and then declined towards the end of the study overall trap types (Figure 6).

### 3.1. Weather Data

**Figure 7:**
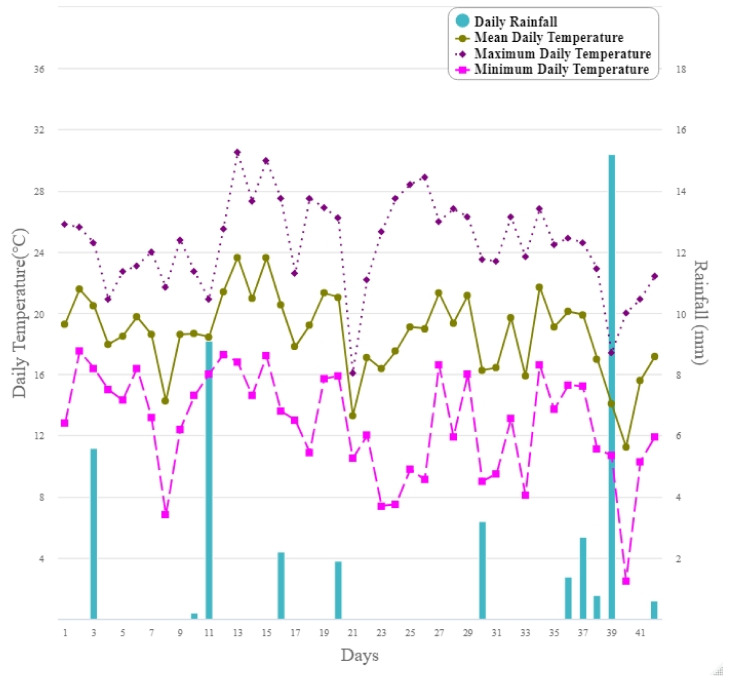
The mean, maximum and minimum temperature (°C) and rainfall (mm) over 42 days (18 January–1 March 2019) from Palmerston North Airport (40°19′15.6″ S latitude, 175°37′05.7″ E longitude) managed by the National Meteorological Service (https://cliflo.niwa.co.nz/, accessed on 1 October 2019) approximately 8 km from the study site.

**Figure 8:**
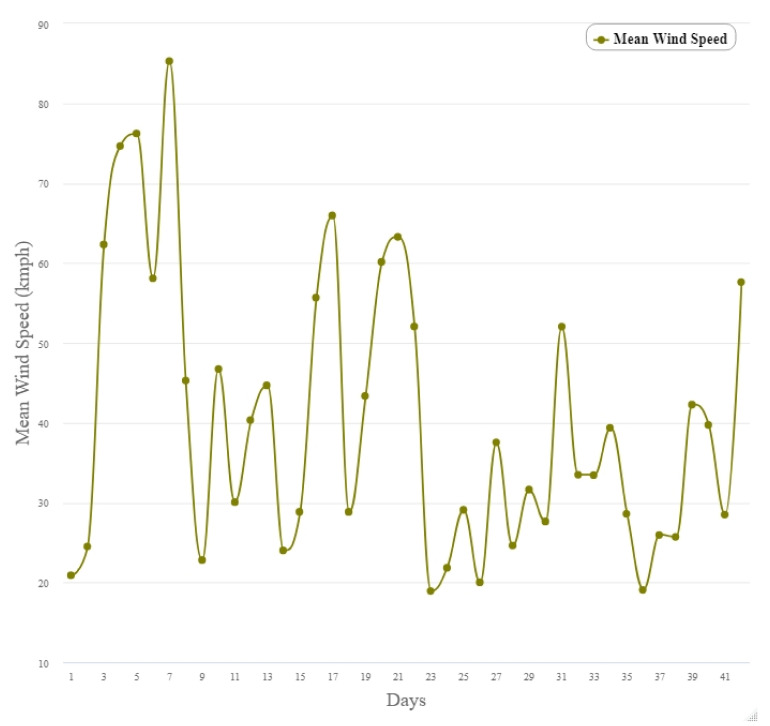
The mean wind speed per day over 42 days (18 January–1 March 2019) from Palmerston North Airport (40°19′15.6″ S latitude, 175°37′05.7″ E longitude) managed by the National Meteorological Service (https://cliflo.niwa.co.nz/, accessed on 1 October 2019) approximately 8 km from the study site.

The weather data illustrated in Figure 7 shows little variability in temperature over the course of the six-week trial period. Rainfall occurred sporadically throughout the study; a total of 42.9 mm fell; with 20.7 mm of this falling in the final week (Figure 7). The maximum temperature for the study period was 28 °C occurring in the second week of the study. The mean and minimum temperatures decreased over the latter three weeks of the study (Figure 7). Windspeed was highest in the first week of the study and sporadically decreased throughout the study (Figure 8).

### 3.2. Results of Mean Model for Lucilia cuprina and Lucilia sericata

#### 3.2.1. *Lucilia cuprina*

**Figure 9:**
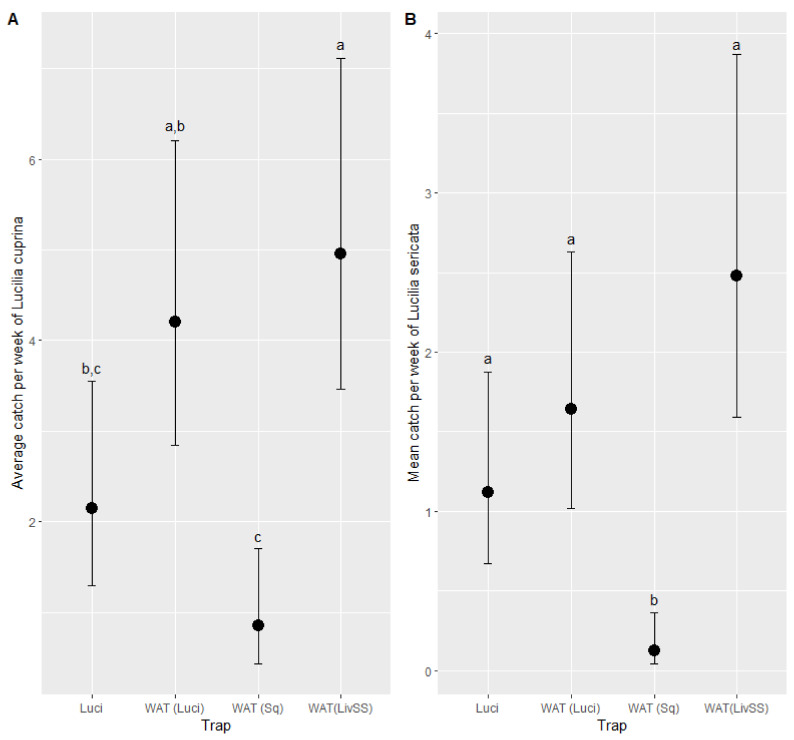
The estimated mean catch per week of *Lucilia cuprina* (**A**) and *Lucilia sericata* (**B**) by each trap type adjusting for the effect of maximum temperature. Trap types with differing letters (a, b, and c), within target species, are significantly different based on a Tukey comparison of least square means ± standard error (*p* < 0.05). LuciTrap with LuciLure A, B and C (Luci); Western Australian Trap with squid (WAT (Sq)), Western Australian Trap with LuciLure A, B and C (WAT (Luci)); and Western Australian Trap with sheep liver and sodium sulphide (WAT (LivSS)).

**Figure A1:**
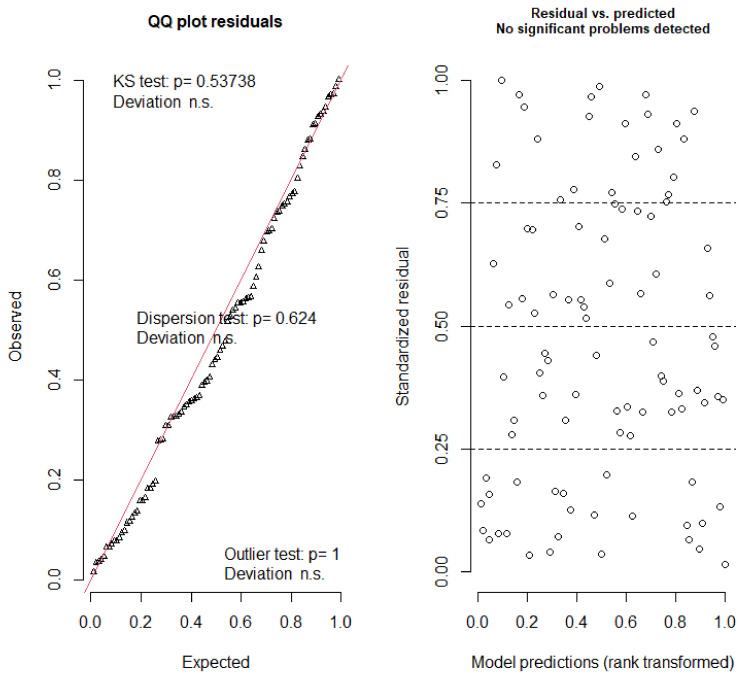
Q-Q plot of and the residuals versus the predicted for the Mean catch model of *Lucilia cuprina*.

The best-fitting model to describe the mean catch of *L. cuprina* was a negative binomial model with an R^2^ of 0.34. In addition to the trap type (Table 4), the maximum temperature was found to be the only weather parameter that was able to significantly (*p* < 0.01) influence the catch of *L. cuprina*. The maximum temperature was found to positively (*p* < 0.05) influence the catch of *L. cuprina*. A Q-Q plot and simulated residuals plotted against observed data both were found to be normally distributed (Figure A1). For *L. cuprina* the highest mean weekly catches were in the WAT (LivSS) and WAT (Luci) with no difference between them (*p* > 0.05) (Figure 9A). Luci caught fewer (*p* < 0.05) than the WAT (LivSS), but no difference (*p* < 0.05) was found between the Luci and the WAT (Luci). There was no difference (*p* > 0.05) between Luci and WAT (Sq) (Figure 9A).

#### 3.2.2. *Lucilia sericata*

**Figure A2:**
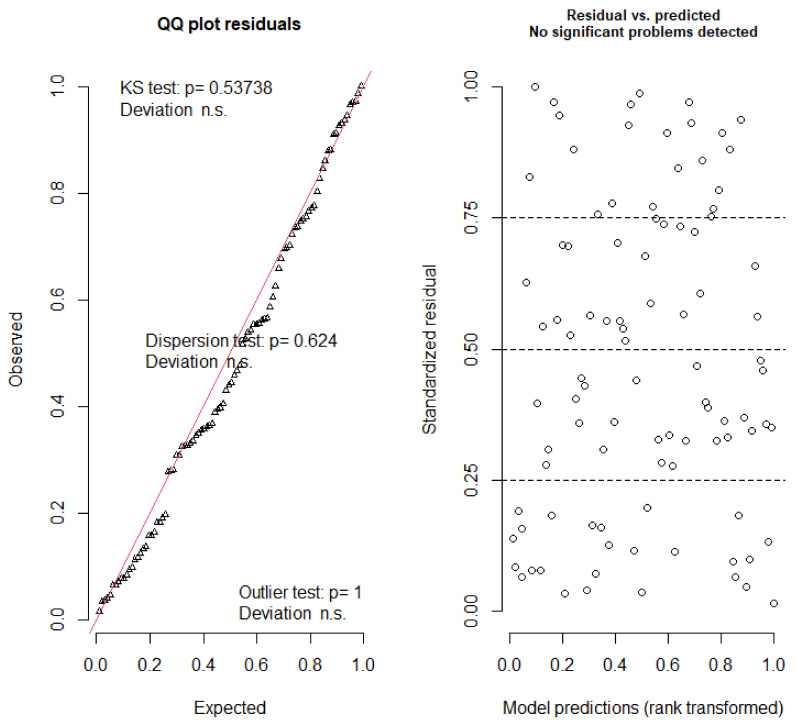
Q-Q plot and the residuals versus the predicted for the mean catch model of *Lucilia sericata*.

The mean catch of *L. sericata* was best described by a negative binomial model with an R^2^ of 0.36. The type of trap and maximum temperature influenced (*p* < 0.05) the catch of *L. sericata* (Table 5). The maximum temperature positively (*p* < 0.05) influenced the catch of *L. sericata*. A Q-Q plot and simulated residuals plotted against observed data both were found to be normally distributed (Figure A2). For *L. sericata*, there were no significant differences (*p* < 0.05) between Luci, WAT (LivSS) and WAT (Luci) (Figure 9B). The WAT (Sq) caught less (*p* < 0.05) than all other trap treatments (Figure 9B).

### 3.3. Results of Rate Model for Lucilia cuprina and Lucilia sericata

#### 3.3.1. *Lucilia cuprina*

**Figure 10:**
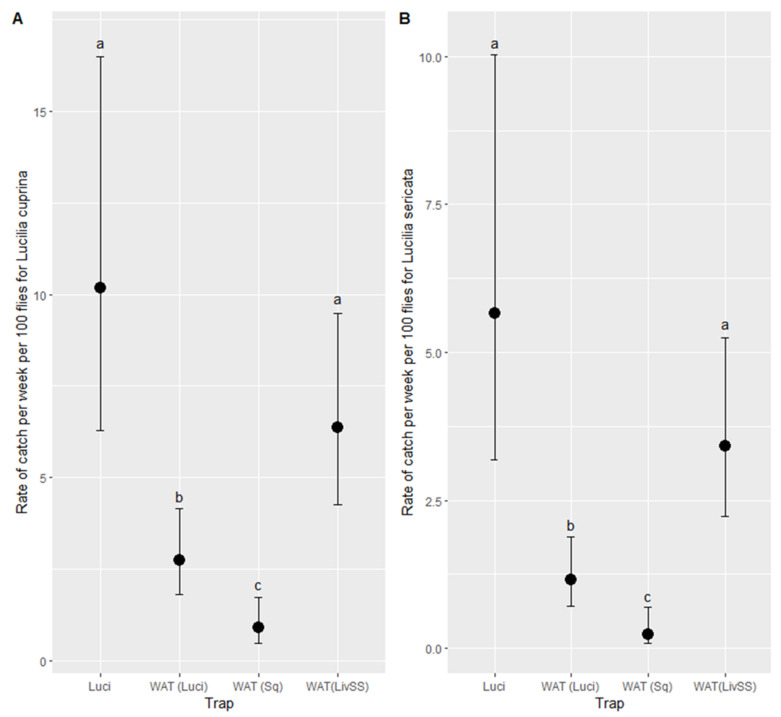
The estimated rate per week of the catch of *Lucilia cuprina* (**A**), adjusting for the effect of rainfall and *Lucilia sericata* (**B**). Trap types with differing letters (a, b, and c), within target species, are significantly different based on a Tukey comparison of least square means ± standard error (*p* < 0.05). LuciTrap with LuciLure A, B and C (Luci); Western Australian Trap with squid (WAT (Sq)), Western Australian Trap with LuciLure A, B and C (WAT (Luci)); and Western Australian Trap with sheep liver and sodium sulphide (WAT (LivSS)).

**Figure A3:**
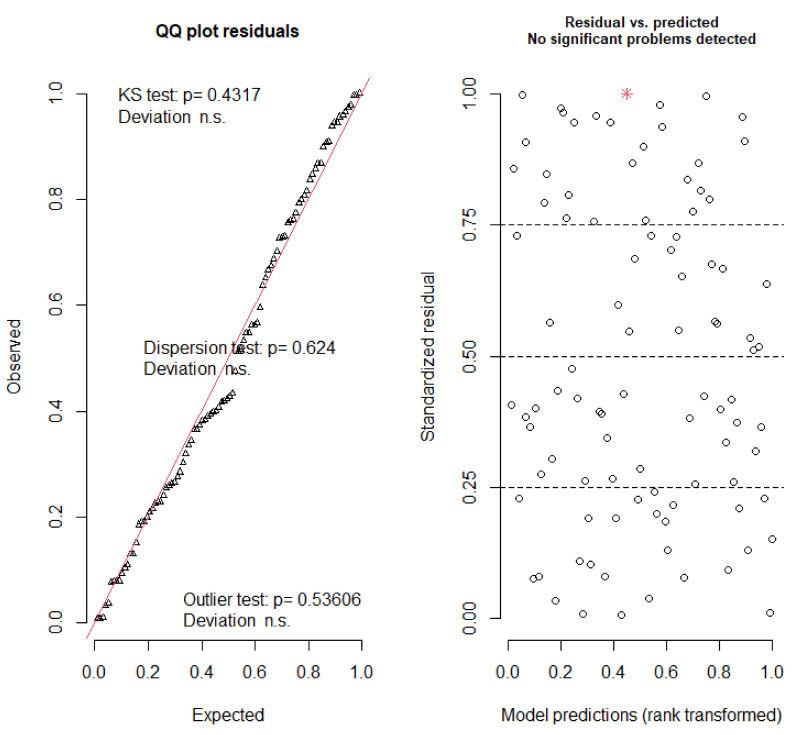
Q-Q plot and the residuals versus the predicted for the rate of catch model of *Lucilia cuprina*.

The best model to describe the rate of catch per week of *L. cuprina* was a negative binomial model with an R^2^ of 0.28. In addition to trap type (Table 6 and Figure 10A), rainfall was found to have a negative effect (*p* < 0.05) (Table 6). The mean and maximum (*p* < 0.05) temperature (*p* > 0.05) did not affect the catch of *L. cuprina*. A Q-Q plot and simulated residuals plotted against observed data both were found to be normally distributed (Figure A3).

#### 3.3.2. *Lucilia sericata*

**Figure A4:**
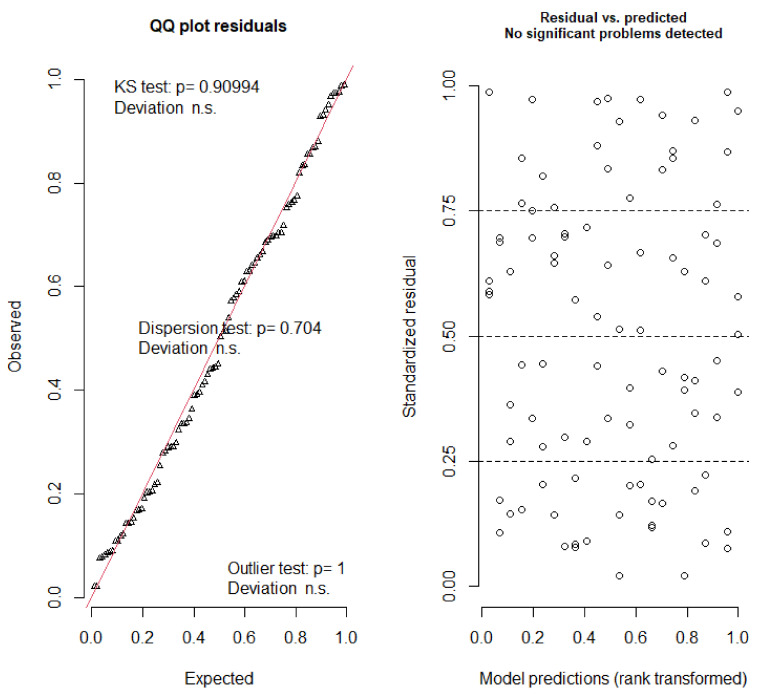
Q-Q plot and the residuals versus the predicted for the rate of catch model of *Lucilia sericata*.

The rate of catch per week for *L. sericata* was best described by a negative binomial model with an R^2^ of 0.35 (Table 7). In addition to the trap type, rainfall had a negative effect (*p* < 0.05), and mean weekly temperature had a positive effect (*p* < 0.05). A Q-Q plot and simulated residuals plotted against observed data both were found to be normally distributed (Figure A4).

The highest rate of catch for both *L. cuprina* or *L. sericata* were with the WAT (LivSS) and the Luci, neither of which were significantly different from each other (*p* > 0.05, Figure 10B) but were both higher than WAT (Luci) (*p* < 0.05), which, in turn, was higher than WAT (Sq) (*p* < 0.05).

## 4. Discussion

The aim of this study was to identify the most efficient trap and bait combination that New Zealand farmers could utilise as part of routine surveillance to monitor the presence of both target species and management of flystrike prevention on farms. Overall, this study found that both the LuciTrap with the LuciLures and the Western Australian Trap with sheep liver and sodium sulphide were the most effective toward *L. cuprina* and *L. sericata*.

The high catch of Muscidae and Sarcophagidae was notable from each of the trap and bait combinations; however, these were not relevant from a flystrike monitoring or control perspective. This study occurred in the mid-late summer period, which is recognised as the mid-season for flystrike target species in New Zealand [1]. The location of the treatments within the Latin square array was not found to have any influence on the fly catch rates or species and, therefore, had no impact on the results found.

In this study, two models were presented to contrast the catch of *L. cuprina* and *L. sericata* by each trap bait combination. The first model estimated the mean catch per week of both target species and the second model estimated the rate of catch per week of the target species by including an offset to adjust for the total catch from each trap each week. Further analysis could not be made in regard to *C. stygia* due to the low sample size, and it is unclear why such a result was obtained.

In the mean catch model, the maximum temperature positively affected both species’ catch. Since temperature has previously been found to be an important environmental variable in regard to the activity of blowflies [22,37,38,39], this result was not unexpected. Dipterans are thermophilic and, therefore, more active in warmer temperatures. Wind was found not to have an effect in either model.

In the rate of catch model, we found that the catch of *L. cuprina* was negatively influenced by rainfall but not by temperature. In contrast for *L. sericata*, the rate of catch was influenced by both rainfall and temperature. This suggests that *L. sericata* may be more sensitive to temperature changes than *L. cuprina* compared to other species in the bycatch. Furthermore, rainfall has also been found to depress the activity of Calliphoridae; thus, it has been linked with lower observed catches in flytraps [39,40]. Interestingly, rainfall had no influence on the mean catch model; the reasoning for this is uncertain.

However, it could be hypothesised that, in the rate of catch model, the effect of rain was on the bycatch, and therefore this changed the rate by having a positive effect on the denominator (total catch) rather than a negative effect on the numerator (target species catch). Otherwise, the effect of rainfall would have been found in the mean model. The negative influence of rainfall on the activity of various Calliphoridae including *L. sericata* has been previously reported in Germany [39]. Whereas, for Sarcophagidae and Muscidae species, climatic variables aside from temperature have not been identified to significantly alter abundance [41,42].

This study indicated that certain trap treatments were better than others for monitoring these target species. In regard to *L. cuprina*, for the mean model, the WAT (LivSS) caught more than the LuciTrap, while, in the rate model, there was no difference between either treatment. These findings do not agree with previous studies in Australia [16,17] where it was found that the LuciLures (i.e., the same treatment as the LuciTrap in the present study) were more selective and attractive for *L. cuprina* than sheep liver in 30% sodium sulphide when using sticky traps.

It was due to the results of these studies that the LuciTrap has been adapted to be the best trap for *L. cuprina*. The differences between the present study and others [16,17] are due to the experimental set-up as the comparisons made in this study are on the basis of the trap and bait type. That being said, it should be noted that the present study is limited to being conducted in one location over a relatively short period of time. Given the results from this study, further variation in different regions with different climates would be expected, whereas the previous Australian studies were conducted across multiple regions over a number of years.

Furthermore, there are fundamental differences in the statistical measures used in this study compared to previous studies [16,17]. Urech, et al. [16,17] log-transformed their data by fly counts +1 in an effort to normalise their data prior to analysis. Our approach was to analyse the results as count data, which recognises the importance of zero counts and their statistical implications.

In contrast for *L. sericata*, both the Luci and the WAT (LivSS) were similar and associated with the highest rate of catch in both types of analysis, indicating that both traps are suitable for monitoring *L. sericata* in New Zealand. The results from this study agree with previous studies [18,21] where *L. sericata* was trapped using the LuciTrap but with a low catch rate. The results of the present study do not agree with a Hungarian study that did not catch any *L. sericata* using the LuciTrap [20]. The difference in the results from this study may be that the LuciTrap was used in the Hungarian study [20] with different bait and not LuciLures. However, this should not be viewed as a critique as there would be considerable logistical difficulties and expenses if one were to import the LuciLures from Australia to Hungary.

In this study, it was proposed that squid could be an effective replacement for either sheep liver and 30% sodium sulphide or the LuciLure for attracting and monitoring the target species in the WA Traps. This would have the added benefit that it would be an easier bait to source and much safer to use on-farm from a health and safety perspective. Squid would also be advantageous as it retains a state of moist decay, and it would remove the need for using chemicals to attract necrophagous flies.

Baz, et al. [43] reported a high success rate for trapping numerous species of Calliphoridae using only squid as bait. However, it was found that the WAT (Sq) had the lowest catch of all traps groups for all target species. Therefore, it is not suitable for this purpose in New Zealand. Although the data from this study would suggest it may be more suitable as bait if the aim was to catch Sarcophagidae and Muscidae. Previous studies [43,44,45] reported that squid as bait caught a wide range of Calliphoridae, Sarcophagidae and Muscidae species.

The combined analysis in the present study indicates that either the WAT (LivSS) or the LuciTrap could be used to catch both *L. sericata* and *L. cuprina* in New Zealand. Given its low bycatch, the LuciTrap makes it far easier for a farmer to quickly identify the presence of either target species. This is particularly important when a farmer is only using a trap to monitor when the first appearance of these target species in a season, so that control procedures can be initiated. While LuciTrap is advantageous given the length of time its chemical bait lasts (i.e., six months), there are legal limitations for the use of its chemicals on farms in New Zealand [24]; which may restrict their future use. This suggests the WAT (LivSS) may be a more acceptable option; however, its use is limited by the need to change the bait weekly, and there are also the same limitations with the use of sodium sulphide on farms in New Zealand [24].

This study appears to be the first report of *Chrysomya megacephala* being caught on a farm in New Zealand. It has previously only been recorded at the New Zealand border [4]. *Chrysomya megacephala* is noted to be a secondary fly strike species that cannot initiate flystrike in the absence of wounds [46]. Therefore, it may be expected that the potential effect on the rate of flystrike by this new species in New Zealand would be negligible.

## 5. Conclusions

The aim of this study was to determine the better trap and bait combination for use on New Zealand farms. The results of this study indicate that the best trap and bait combinations of those tested were the LuciTrap with its LuciLures or the WAT (LivSS) for monitoring either *L. cuprina* or *L. sericata*. Overall, a clear difference in the catch of Calliphoridae was identified between the three treatments that had chemicals (LuciTrap, WAT (Luci) and WAT (LivSS) and the squid bait.

Future studies should consider the effectiveness of combinations of sheep liver and/or other types of offal with other fish baits to attract *Lucilia* as alternatives to using chemical baits. In addition, future studies could also consider contrasting these non-chemical baits with aged offal in a similar manner to this study. The adaptation of the most effective treatments from this study on New Zealand farms may also be limited by the current legislation regarding the use of chemicals on farms.

## Figures and Tables

**Table 1:** Summary of the trap and bait combinations used in this study.

Trap Type	Bait Type	Acronym
LuciTrap	LuciLure baits *	Luci
Western Australian Trap	50 g of squid	WAT (Sq)
Western Australian Trap	LuciLure baits *	WAT (Luci)
Western Australian Trap	50 g of sheep liver in 100 mL of 30% sodium sulphide	WAT (LivSS)

* LuciLures include lures A, B and C [15,16,17,19].

**Table 2:** The total number and species of flies caught over six weeks by Luci ^a^; WAT (Sq) ^b^; WAT (Luci) ^c^; and WAT (LivSS) ^d^ trap types.

Order	Family	Species	Luci ^a^	WAT(Sq) ^b^	WAT (Luci) ^c^	WAT (LivSS) ^d^	Total
Diptera	Calliphoridae	*Lucilia cuprina*	70	15	125	151	361
		*Lucilia sericata*	39	4	61	84	188
		*Calliphora stygia*	0	0	2	1	3
		*Calliphora quadrimaculata*	0	1	0	1	2
		*Calliphora vicina*	1	0	0	0	1
		*Chrysomya rufifacies*	0	1	0	9	10
		*Chrysomya megacephala*	0	0	0	2	2
	Fanniidae		7	2	2	0	11
	Sarcophagidae		1502	3559	7224	5928	18,213
	Muscidae		326	850	1401	1170	3747
	Polleniiidae		23	5	2	4	34
	Staphylinidae		0	0	2	0	2
	Unknown		4	2	0	1	7
Hymenoptera	Vespidae		0	0	0	1	1
Total			1974	4438	8823	7381	22,616

**^a^** LuciTrap with LuciLure A, B and C. ^b^ Western Australian trap with squid. ^c^ Western Australian trap with LuciLure A, B and C. ^d^ Western Australian trap with sodium sulphide and sheep liver.

**Table 3:** The mean (95% confidence intervals, (CI)) unadjusted weekly catch of *Lucilia cuprina*, *Lucilia sericata* and by-catch in Luci ^a^; WAT (Sq) ^b^; WAT (Luci) ^c^; and WAT (LivSS) ^d^ trap types.

Trap	*L. cuprina*	*L. sericata*	By-Catch ^e^
Luci ^a^	11.7 (1.4–21.9, 95% CI)	6.5 (1.4–11.6, 95% CI)	310.8 (26.2–595.5, 95% CI)
WAT (Sq) ^b^	2.5 (1.4–3.6, 95% CI)	0.7 (0.1–1.2, 95% CI)	736.5 (82.8–1390.2, 95% CI)
WAT (Luci) ^c^	20.8 (7.7–33.9, 95% CI)	10.2 (3.2–17.1, 95% CI)	1439.5 (605.8–2273.2, 95% CI)
WAT (LivSS) ^d^	25.5 (7.0–44, 95% CI)	14.0 (3.2–17.1, 95% CI)	1191.0 (93.2–2288.8, 95% CI)

^a^ LuciTrap with LuciLure A, B and C. ^b^ Western Australian trap with squid. ^c^ Western Australian trap with LuciLure A, B and C. ^d^ Western Australian trap with Sodium Sulphide and sheep liver. ^e^ By-catch included all non-target species.

**Table 4:** Summary of the model coefficients, confidence intervals (CI) and *p* values for each predictor of mean catch of *Lucilia cuprina*. The mean catch model includes the catch of the target species and the catch of all species in each trap per week.

Predictors	Co-Efficients	CI	*p* Value
Intercept	−7.28	−12.55–−2.01	0.007
WAT (LivSS)	reference	-	-
Luci ^a^	−0.84	−1.39–−0.28	0.003
WAT (Sq) ^b^	−1.75	−2.45–−1.05	<0.001
WAT (Luci) ^c^	−0.17	−0.63–0.29	0.478
Week ^d^	0.49	0.26–0.72	<0.001
Maximum Temperature ^e^	0.26	0.10–0.42	0.002

^a^ LuciTrap with LuciLure A, B and C. ^b^ Western Australian trap with squid. ^c^ Western Australian trap with LuciLure A, B and C. ^d^ Week was defined as the week of catch. ^e^ Maximum Temperature was designated as the maximum temperature each week.

**Table 5:** Summary of model coefficients, confidence intervals (CI) and *p* values for each predictor of mean catch of *Lucilia sericata*. The mean catch model includes the catch of the target species and the catch of all species in each trap per week.

Predictors	Co-Efficients	CI	*p* Value
Intercept	−10.93	−17.42–−4.43	0.001
WAT (LivSS)	reference	-	-
Luci ^a^	−0.79	−1.41–−0.18	0.012
WAT (Sq) ^b^	−3.00	−4.11–−1.89	<0.001
WAT (Luci) ^c^	−0.41	−1.00–0.17	0.165
Week ^d^	0.62	0.33–0.90	<0.001
Maximum Temperature ^e^	0.35	0.15–0.55	0.001

^a^ LuciTrap with LuciLure A, B and C. ^b^ Western Australian trap with squid. ^c^ Western Australian trap with LuciLure A, B and C. ^d^ Week was defined as the week of catch. ^e^ Maximum Temperature was designated as the maximum temperature each week.

**Table 6:** Summary of model coefficients, confidence intervals and *p* values for each predictor of the rate of catch per week of *Lucilia cuprina*. The rate of catch per week model included an offset of the bycatch (i.e., all other flies other than *L. cuprina* and *L. sericata*) in each trap.

Predictors	Co-Efficients	CI	*p* Value
Intercept	−5.19	−5.93–−4.44	<0.001
WAT (LivSS)	Reference	-	-
Luci ^a^	0.47	−0.13–1.07	0.127
WAT (Sq) ^b^	−1.96	−2.71–−1.21	<0.001
WAT (Luci) ^c^	−0.84	−1.40–−0.27	0.004
Week ^d^	0.52	0.35–0.69	<0.001
Rainfall ^e^	−0.04	−0.07–0.00	0.043

^a^ LuciTrap with LuciLure A, B and C. ^b^ Western Australian trap with squid. ^c^ Western Australian trap with LuciLure A, B and C. ^d^ Week was defined as the week of catch. ^e^ Rainfall was designated at the total rainfall each week.

**Table 7:** Summary of model coefficients, confidence intervals and *p* values for each predictor of the rate of catch per week of *Lucilia sericata*. The rate catches per week model included an offset of the bycatch (i.e., all other flies other than *L. cuprina* and *L. sericata*) in each trap.

Predictors	Co-Efficients	CI	*p* Value
Intercept	6.06	−10.19–22.30	0.465
WAT (LivSS)	reference	-	-
Luci ^a^	0.50	−0.19–1.19	0.153
WAT (Sq) ^b^	−2.67	−3.80–−1.54	<0.001
WAT (Luci) ^c^	−1.09	−1.71–−0.47	0.001
Week ^d^	0.30	−0.13–0.74	0.172
Rainfall ^e^	−0.08	−0.14–−0.01	0.019
Mean Temperature ^f^	−0.58	−1.35–0.20	0.145

^a^ LuciTrap with LuciLure A, B and C. ^b^ Western Australian trap with squid. ^c^ Western Australian trap with LuciLure A, B and C. ^d^ The week was defined as the week of catch. ^e^ Rainfall was designated at the total rainfall each week. ^f^ Mean Temperature was designated as the Mean Temperature each week.

## Data Availability

Data is available on request from the corresponding author.

## Appendix A

The following are the diagnostic results of each of the models from this study.
